# Identification of mRNAs differentially-expressed between benign and malignant breast tumour cells

**DOI:** 10.1038/sj.bjc.6600456

**Published:** 2002-08-12

**Authors:** D Liu, P S Rudland, D R Sibson, R Barraclough

**Affiliations:** School of Biological Sciences, Life Sciences Building, University of Liverpool, PO Box 147, Liverpool L69 7ZB, UK; Clatterbridge Cancer Research Trust, JK Douglas Laboratories, Clatterbridge Hospital, Bebington, Wirral CH63 4JY, UK

**Keywords:** suppression subtraction hybridisation, oestrogen receptor, chromosome 21, breast cancer

## Abstract

Two suppression subtracted cDNA libraries have been constructed, one containing cDNAs to mRNAs present at a higher level in a benign human breast tumour-derived cell line relative to the malignant mammary cell line, MCF-7, and the other containing cDNAs present at a higher level in the MCF-7 cells relative to the benign cells. Randomly-picked cloned DNAs have been sequenced yielding 29 and 128 different cDNAs from the benign and malignant libraries, respectively. Using reverse Northern hybridisation, 76% and 83% of the cDNAs were differentially expressed by greater than two-fold, whilst 14% and 11% of cDNAs in the respective libraries were differentially expressed by more than 15-fold. Amongst these were oestrogen-responsive cDNAs and expressed sequence tags. One such oestrogen-responsive expressed sequence tag, M41, is transcribed from a gene located on chromosome 21q22.3, within an intron of a larger gene. The M41 gene contains oestrogen response elements, one of which is associated with *alu* repeats. M41 mRNA is expressed at a statistically significantly higher level in human breast cancer specimens than in normal human breast and benign lesions. In carcinomas, its up-regulation is associated with the development of the malignant cell.

*British Journal of Cancer* (2002) **87**, 423–431. doi:10.1038/sj.bjc.6600456 www.bjcancer.com

© 2002 Cancer Research UK

## 

Cancer in the Western world affects one in three of the population, and it is the malignant properties of invasion and metastasis that are harmful to the patient. Many cancers are thought to arise from tumours, which progress from a benign state to a malignant one (Nowell, 1986). The benign tumours can arise by a variety of mechanisms including changes in gene expression and mutation of key growth regulatory genes; however, the progression of a tumour to a life-threatening malignant state involves more-widespread changes in gene expression (Fearon and Vogelstein, 1990). Thus, the difference between the behaviour of benign tumour cells and cancer cells is likely to be explained, at least in part, by identifying the genes and gene products which are differentially expressed between them (Martin *et al*, 2000). Such an approach has yielded the metastasis-inducing protein S100A4 (Davies *et al*, 1993; Barraclough, 1998). In order to identify a wider range of mRNAs encoding protein products which might contribute to the progression of oestrogen receptor positive breast tumour cells, PCR -selected suppression subtractive hybridisation has now been employed to construct subtracted libraries in alternative orientations using mRNAs isolated from a benign human mammary epithelial cell line, Huma 123 (Ke *et al*, 1993), and from the malignant human mammary epithelial cell line, MCF-7. The resulting subtracted libraries not only contain previously-characterised differentially-expressed cDNAs associated with the presence of oestrogen receptor, but also contain novel cloned cDNAs which may also be associated with the development of breast cancer.

## MATERIALS AND METHODS

### Human breast cell lines

Huma 7, a normal human mammary epithelial cell line that had been immortalised with SV40 virus (Rudland *et al*, 1989), Huma 123, a benign human mammary epithelial cell line, and Huma 109, a benign human mammary elongated myoepithelial-like cell line, derived from a primary cell culture of human fibrocystic disease displaying prominent epithelial hyperplasia (Briand *et al*, 1987; Ke *et al*, 1993), and the malignant human mammary epithelial cell lines, MCF-7, T47D, and ZR-75-1, derived from pleural effusions of breast cancer patients (Soule *et al*, 1973; Engel and Young, 1978) and MDA-MB-231 (Cailleau *et al*, 1974) were propagated in a humidified atmosphere of 95% air, 5% CO_2_ at 37°C as described below. The Huma 7 cell line was grown in DMEM, 5% (v v^−1^) foetal calf serum (FCS), 50 ng ml^−1^ each of insulin and hydrocortisone. The benign cell lines were grown in 50% DMEM, 50% RPMI 1640 medium, 5% (v v^−1^) FCS, 50 ng ml^−1^ insulin, 5 ng ml^−1^ EGF (Rudland *et al*, 1989; Ke *et al*, 1993). Cell line MCF-7 was propagated in DMEM, 5% (v v^−1^) FCS, 50 ng ml^−1^ insulin, 10^−8^ M oestradiol; T47D in DMEM, 10% (v v^−1^) FCS, 1 μg ml^−1^ insulin, 2.5 ng ml^−1^ EGF; ZR-75-1 in DMEM, 5% (v v^−1^) FCS, 2.5 ng ml^−1^ EGF, 10^−8^ M oestradiol. The MDA-MB-231 cell line was propagated in DMEM, 5% (v v^−1^) FCS, 50 ng ml^−1^ insulin, 2.5 ng ml^−1^ EGF. Media used for growing cells contained phenol red. Cell lines were grown in the same batch of foetal calf serum and harvested when they reached 70% confluence. MDA-MB-231 cells were growing slightly faster than Huma 7, Huma 109, MCF-7, T47D, ZR-75-1, and Huma 123 were growing slightly more slowly than the others. Broadly, however, the cells were growing at a similar rate.

The Huma 123 cells, used for the construction of the subtracted libraries, are a unique single-cell cloned line derived from a primary culture of human benign fibrocystic disease. The cell line has been characterised as a breast epithelial cell by immunochemical detection of breast epithelial-associated antigens, epithelial membrane antigen, milk fat globule membrane antigen and keratin 18 in cell culture and in tumours produced in immunosuppressed *nu-nu* mice. In culture, the cells are diploid both in culture and when growing in tumours in *nu-nu* mice. They possess regular nuclei with little or no pleomorphism, consistent with a nonmalignant morphology. The tumours are encapsulated and show no evidence of local invasion or dissemination. Huma 123 cells thus have the characteristics of a neoplastic but not malignant phenotype (Ke *et al*, 1993; Rudland, 1993). The Huma 123 cell line has not been manipulated in order to make it immortal for culture purposes. Since it is difficult to obtain cultures of normal/benign human breast epithelial cells, the Huma 123 cell line is uniquely suited for comparison with more malignant cells for studies into the early stages of the development of malignancy.

### Construction of subtracted cDNA libraries between human breast tumour cell lines, Huma 123 and MCF-7

Suppression subtracted libraries were constructed using a PCR-Select™ cDNA Subtraction Kit (Clontech, Palo Alto, CA, USA), according to the manufacturer's methodology. Critical steps of the construction of the libraries were checked before proceeding to the next step. Double-stranded cDNAs of both tester and driver were synthesised from 2 μg poly(A)^+^-containing RNA derived from the cultured benign cell line, Huma 123 and the malignant cell line MCF-7. The efficiencies of double-stranded cDNA synthesis and digestions with restriction enzyme *Rsa*I were monitored by agarose gel electrophoresis. The two portions of fragmented ds cDNAs of tester, ligated with different specific adaptors (1 and 2) were subjected to hybridisation with the fragmented ds cDNA of driver. After suppression PCR amplification, the resulting subtracted PCR pools of benign or malignant subtracted libraries ranged in size from 200 bp to 1 kbp. The PCR products constituting the subtracted libraries were ligated into a ddT-tailed pBluescript vector, and transformed into *E. coli* XL-1 blue bacteria by electroporation, followed by plating on agar containing ampicillin, IPTG and X-gal. More than 90% of the randomly-picked clones contained cDNA inserts.

### DNA sequencing analysis of the subtracted libraries

Nucleic acid sequences of cDNA clones from the benign and malignant subtracted libraries were determined using an automated ABI 377 DNA sequencing system and standard dye terminator AmpliTaq kits (Perkin Elmer, Buckinghamshire, UK). The resulting DNA sequences were analysed for homology using the public GenBank/EMBL/DDBJ/PDB and Expressed Sequence Tag (EST) databases using the Basic Local Alignment Search Tool (BLAST) program (http://www.ncbi.nlm.nih.gov/BLAST) (Altschul *et al*, 1990). The DNA sequences were further analysed using the GCG program (University of Wisconsin, WI, USA).

### Differential screening of the subtracted libraries using reverse Northern blot

The cloned cDNA inserts were amplified directly from individual colonies of both subtracted benign and malignant libraries using colony PCR. After amplification, PCR products were denatured and spotted onto duplicate nylon membranes using a slot- or dot-blot apparatus. The filters were hybridised with equal concentrations of double-stranded cDNA derived from driver and tester mRNA, respectively, which had been labelled to approximately equal specific activity (1×10^9^ d.p.m μg^−1^ DNA) with ^32^P-dCTP as described previously (Feinberg and Vogelstein, 1984).

### RNA electrophoresis and Northern hybridisation

Gel electrophoresis of RNA in formaldehyde denaturing gels and Northern blotting were performed according to standard procedures (Sambrook *et al*, 1989). The filters were hybridised with equivalent amounts of cloned double-stranded cDNA labelled as above. The intensities of the autoradiographic images were quantified using IMAGE software (NIH, Bethesda, MD, USA).

### Rapid amplification of cDNA ends for cloning of full length cDNA

Cloning of full length M41 cDNA was carried out by rapid amplification of cDNA ends (RACE) using a Marathon cDNA amplification Kit (Clontech) on 2 μg of poly(A)^+^-containing RNA derived from the MCF-7 cell line. The resulting adaptor-ligated ds cDNA, which represented an uncloned ds cDNA library, was used to perform 5′ and 3′ RACE reactions with the following M41 gene specific primers for 5′ and 3′ RACE reactions respectively: (5′-CCTCACGCTGTCTGGTTGGCTTTCC-3′), (5′-GGTATGCAGCTGATAAGACGCTATAGAG-3′), based on the isolated M41 EST sequence.

### Human breast tissue, immunocytochemistry and *in situ* hybridisation

Normal and benign human breast specimens and invasive ductal carcinomas were obtained from the Liverpool Cancer Tissue Bank Research Centre and the Royal Liverpool University Hospital (Liverpool, UK) with legal consent as described previously (Taylor *et al*, 1998). Immunocytochemical detection of oestrogen receptor α (Platt-Higgins *et al*, 2000), *in situ* hybridisation and PCR reactions (Liu *et al*, 2000) were carried out as described previously.

## RESULTS

### Construction of subtracted cDNA libraries

Libraries of cDNAs from the benign Huma 123 cells, suppression subtracted with cDNA from MCF-7 cells (benign library), and from the MCF-7 cells suppression subtracted with cDNA from the Huma 123 cells (malignant library) were constructed. The subtraction procedure reduced the abundancies of glyceraldehyde-3-phosphate dehydrogenase (GAPDH) cDNA in the malignant and benign subtracted libraries about 36-fold and 40-fold, respectively, relative to their abundancies in the unsubtracted cDNAs, as indictated by Southern hybridisation of PCR-amplified GAPDH cDNA.

Overall, 87% of the randomly-picked cloned cDNAs yielded sequence information. 128 sequences (comprising 72 different known cDNAs, 53 expressed-sequence-tags (ESTs) and three unmatched sequences) and 29 sequences (comprising 19 separate known cDNAs, eight ESTs and two novel sequences) were obtained from 174 and 52 sequenced cDNA clones from the malignant and benign subtracted libraries, respectively.

### Differential screening of the subtracted libraries using reverse Northern hybridisation

The relative levels between the Huma 123 and MCF-7 cells of the mRNAs corresponding to the sequenced cloned cDNAs, isolated from the subtracted benign and malignant libraries, were estimated by reverse Northern hybridisation, using as probes, cDNA produced from mRNA from either the Huma 123 or MCF-7 cells. The hybridisation results were normalised using cDNAs corresponding to mRNAs for 36B4 (Laborda, 1991) and glyceraldehyde-3-phosphate dehydrogenase (GAPDH), both of which show similar expression between the Huma 123 and MCF-7 cell lines. Using an expression ratio of over two-fold as cut-off, 22 of the 29 cDNA clones, and 99 of 119 cDNA clones examined by reverse Northern screening were identified in the benign and malignant subtracted libraries, respectively (Table 1

**Table 1:**
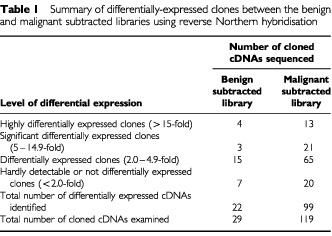
Summary of differentially-expressed clones between the benign and malignant subtracted libraries using reverse Northern hybridisation

). The identities of these cDNAs are shown in Table 2

**Table 2:**
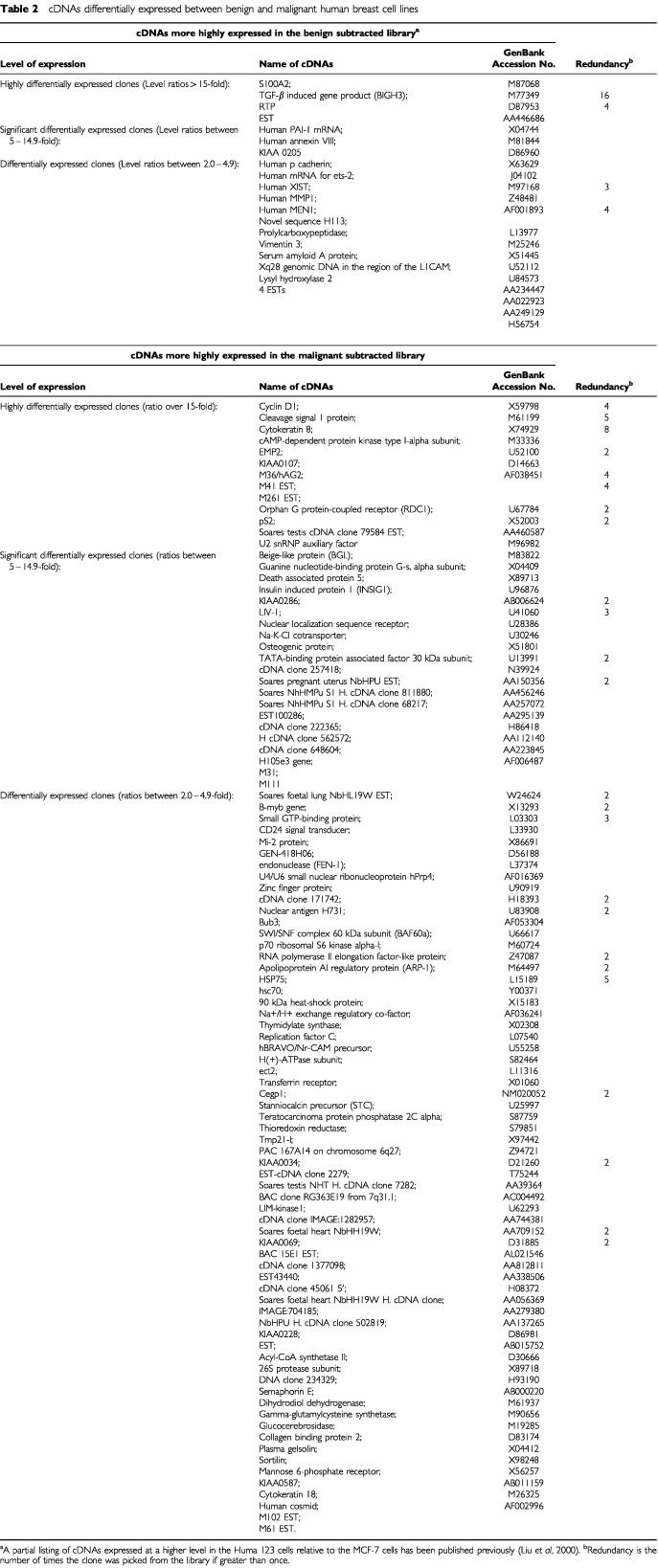
cDNAs differentially expressed between benign and malignant human breast cell lines

. The fact that these totals of cDNAs differentially-expressed by more than two-fold represent respectively 76% and 83% of the total cDNAs examined shows that the subtractive hybridisation procedure was yielding predominantly differentially expressed cDNAs.

Four and 13 cDNAs were differentially expressed by over 15-fold in the benign and malignant breast tumour cell lines, respectively (Tables 1 and 2). Amongst the cDNAs expressed at a higher level in the MCF-7 cells than in the Huma 123 cells were previously characterised oestrogen-responsive genes, including pLIV-1 (Manning *et al*, 1988) (nine-fold), pS2 (Masiakowski *et al*, 1982) (20-fold) and Cyclin D1 (Altucci *et al*, 1996) (15-fold), consistent with the malignant breast cancer-derived MCF-7 cell line being immunocytochemically positive, and the benign Huma 123 cell line being immunocytochemically negative, for oestrogen receptor α (not shown).

### Clone M41

**Figure 1:**
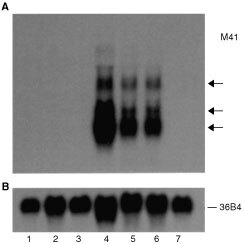
Occurrence of M41 mRNA in human breast tumour cell lines. Total RNAs were isolated from the SV40-immortalised normal human mammary cell line, Huma 7 (lane 1), an elongated converted cell line, Huma 109 (lane 2) derived from the benign mammary tumour-derived cell line Huma 123 (lane 3), the malignant mammary epithelial cell lines MCF-7 (lane 4), T47-D (lane 5), ZR-75 (lane 6) and MDA-MB-231 (lane 7). These RNAs were subjected to Northern hybridisation as described in Materials and Methods, using a ^32^P-labelled probe to M41 mRNA (**A**) or to ribosomal phosphoprotein, 36B4, mRNA (**B**) to check for the loading of RNA onto the gel. The arrows point to three bands of hybridisation in the three oestrogen receptor positive human breast cancer cell lines.

Clone M41, corresponded to an expressed sequence tag that represented an mRNA that was expressed at a 17-fold higher level in the mRNA from the MCF-7 cells than in the Huma 123 cells (Table 2). In Northern hybridisation experiments, the M41 probe hybridised to a major band of RNA of 0.9–1.3 kb from the MCF-7 cells, but there were two additional, but fainter bands of hybridisation at 1.8±0.1 kb and 3.8±0.1 kb (Figure 1

**Figure 3:**
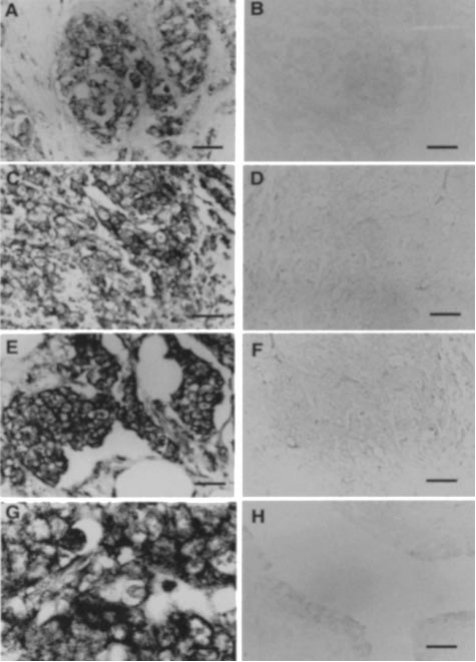
*In situ* hybridisation of M41 mRNA in human breast tumours. Histological sections of breast carcinoma specimens (**A**–**G**), and of one benign tumour specimen (**H**) were subjected to *in situ* hybridisation using antisense (**A**, **C**, **E**, **G**, **H**) or sense (**B**, **D**, **F**) probes to M41 mRNA as described in Materials and Methods. The carcinoma cells in the specimens in **A**, **C**, **E** and **G** stain with an antisense probe of M41 cRNA, whereas there was no staining of adjacent sections of the specimens with sense M41 RNA probe (**B**, **D, F**). (**H**) shows an example of a fibroadenoma which did not stain with an antisense probe, in common with four other fibroadenomas tested. Magnification is ×231 (**A–D**, **F**) or ×578 (**E**, **G**, **H**). Bars=43 μm (**A**–**D**, **F**) or 17 μm (**E**, **G**, **H**).

). M41 mRNA was abundantly expressed in RNA from oestrogen receptor-positive mammary cell lines, T47-D, ZR-75, but was completely undetectable in oestrogen-receptor-negative cell lines, the malignant human mammary cell line, MDA-MB-231 and the benign mammary-derived cell lines Huma 123. M41 mRNA was also undetectable in the normal-derived and benign human mammary epithelial cell lines, Huma 7 and Huma 109 (Figure 1).

**Figure 2:**
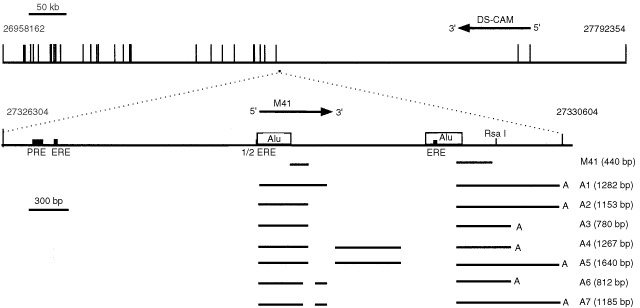
Alignment of the M41 gene and M41 transcripts. The gene region of chromosome 21q22.1 is shown at the top with the exons of the DS-CAM gene shown as vertical bars. The location of the M41 gene is shown as a square to scale beneath the genomic DNA and expanded beneath. The location of response elements for oestrogen (ERE) and progesterone (PRE) and *Alu* sequences are shown. The region of the original M41 cDNA isolated from the MCF-7 subtracted library, and processing variants of M41 mRNA (A1–A7) obtained from the RACE reactions are shown as horizontal bars with gaps indicating the intronic regions. Numbering refers to the base number of contig NT_011512.3. The 5′ intron exon boundaries revealed by the RACE reactions at 27328651, 27328698, 27328827, 27329381, and the 3′ intron exon boundaries at 27328749, 27328894, 27329821 (enumeration of contig NT_011512.3) all contained conserved GT / AG sequences and exhibited 62.5, 75, 87.5, 100, 75, 93.75, and 81.25% identity, respectively, to the consensus sequences for 5′ or 3′ intron exon boundaries (Mount, 1982). The symbols (A) at the ends of the lines indicate the poly(A) addition sites. Variants contained one of two alternative poly(A) addition sites (at 27330601 and 27330228 of NT_011512.3), with a consensus AATAAA poly(A)-addition signal, 29 and 19 nucleotides upstream of the poly(A)-addition sites, respectively. The lengths of the proposed mRNA variants arising from the 7 alternatively-spliced exons correspond broadly to the sizes of two of the bands observed in the Northern blot for the mammary tumour cell line MCF-7 (Figure 1), strongly suggesting that the RACE products are defining near full length mRNAs. Horizontal arrows indicate the direction of transcription of the genes.

The sequence of M41 is located on the human genomic contig GenBank NT_011512.3, which contains 28515322 bp of cloned DNA from chromosome 21q22.3. Using RACE reactions to identify 5′ and 3′ regions of the M41 mRNA, 3′ RACE yielded 10 separate products, each of which corresponded to one of four patterns, which precisely matched the NT_011512.3 genomic DNA sequence to the poly(A)^+^ addition sequence. 5′-RACE yielded 16 cloned sequences, each of which corresponded to one of seven patterns, which matched precisely the NT_011512.3 genomic DNA sequence. Taken together these results identified at least seven experimentally-determined alternatively-spliced transcripts of M41 mRNA in the MCF-7 RNA (M41, A1-7, GenBank accession numbers : AF401029, AF401030, AF401031, AF401032, AF401033, AF401034, AF401035) containing up to three introns (Figure 2

).

Genomic DNA in the region of M41 in contig NT_011512.3 contained two *Alu* repeat sequences, 264 bp and 286 bp in length respectively, located between 27328305 and 27328568 and between 27329569 and 27329854, upstream and adjacent to two of the proposed exons of the M41 gene. There are two oestrogen-response-elements (ERE): AGGTCA-N_23_-TGACCT and AGGTCA-N_17_-TGACC and one half ERE located between 27326764 and 27326797, 27328299 and 27328305, and between 27329636 and 27329663, of contig NT_011512.3 sequence (Figure 2), consistent with the abundance of M41 mRNA in the ER positive cells lines (Figure 1).

The gene for M41 does not presently occur in the annotated human genome sequence. Thus, mapping of contig NT_011512.3 onto the published complete GenBank sequence of human chromosome 21 placed the M41 sequence between nucleotides 40864055 bp and 40866330 bp, located between existing known genes, PCP4 at 40348176 bp and BACE2 at 41649179 bp (Table 3

**Table 3:**
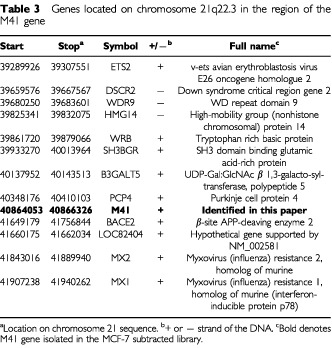
Genes located on chromosome 21q22.3 in the region of the M41 gene

) at a previously unmapped location. However, contig NT_011512.3 also contains part of a previously described gene, Down Syndrome Cell Adhesion Molecule (DS-CAM, cDNA accession AF023450; protein accession, AAC17967) on the complementary strand in the region of the M41 gene. The M41 coding region is located on the complementary strand within the 323 kbp intron 2 of this gene (Figure 2).

**Figure 4:**
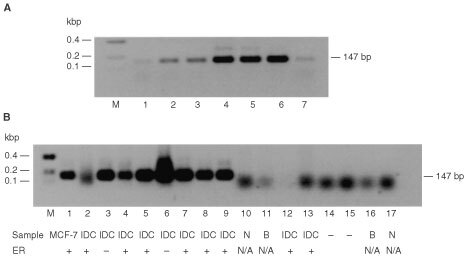
Occurrence of M41 mRNA in benign and malignant human mammary cell lines and breast tumour specimens by RT–PCR. Total RNA from human mammary cell lines. Normal breast derived Huma 7 (**A**, lane 1), benign breast derived Huma 123 (lane 2), Huma 109 (**A**, lane 3), and breast carcinoma-derived cell lines, MCF-7 (**A**, lane 4), T47-D (**A**, lane 5), ZR-75 (**A**, lane 6), MDA-MB-231 (**A**, lane 7), and total RNA from breast carcinoma specimens (**B**, lanes 2–9, 12–13), normal breast specimens (**B**, lanes 10 and 17), and benign breast specimens (**B**, lanes 11 and 16) were amplified by RT–PCR using primers specific for M41 yielding PCR products of 147 bp. (**B**), lanes 14 and 15 are the negative RT–PCR controls, and lane 1 is MCF-7 cell line RNA as positive control. The resulting RT–PCR products were subjected to agarose gel electrophoresis and stained with ethidium bromide, as described in Materials and Methods. Lane M, DNA molecular weight markers. The estrogen receptor status of the specimens is shown beneath panel B as +, positive or -, negative. N/A is not available. The faint bands in **A**, lanes 1–3 and 7 arise from the amplification of a low, otherwise undetectable, level of M41 mRNA in these specimens. The diffuse bands in **B**, lanes 2, and 10–17 at <100 bp are caused by primer dimers. DNA sequencing of the 147 bp band confirmed its identity with the expected sequence of the amplified band.

In order to find out whether M41 mRNA is expressed in human tumour specimens, *in situ* hybridisation was carried out (Figure 3) using sense and antisense probes derived from the region of the M41 clone spanning two exons (Figure 2). Whereas there was no hybridisation to any of the specimens with the sense probe, the antisense probe yielded strong hybridisation in the carcinoma cells of 10 carcinoma specimens and three specimens were negative. There was no statistically significant correlation between M41 positivity and oestrogen receptor positivity for these samples (*P*=1.00, two-tailed Fisher Exact test). In contrast, there was no staining using the antisense probe on a benign fibroadenoma (Figure 3), nor on four other fibroadenoma specimens examined. There was a statistically significant difference between the benign and malignant samples for M41 mRNA positivity (*P*=<0.007, two-tailed Fisher Exact test). Using a sensitive reverse transcript PCR (RT–PCR) assay which yielded an M41-specific 147 bp product (Figure 4

), RNA from 16 normal/benign, and 26 malignant breast tumour specimens were screened for the presence of M41 mRNA (Table 4

**Table 4:**
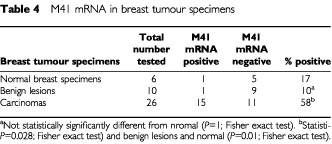
M41 mRNA in breast tumour specimens

). Although there was no correlation at all between the presence of RT–PCR detectable M41 mRNA-specific PCR product and oestrogen receptor positivity (*P*=1.00, Fisher exact test) in the breast carcinoma specimens, M1 mRNA was detectable in 15 out of 26 malignant lesions, but there was an amplified product in only one out of five normal and one out of nine benign breast specimens. There was a strong statistically-significant difference between the occurrence of M41 mRNA in carcinoma specimens and its occurrence in normal/benign specimens (*P*=0.01, two-tailed Fisher exact test) or benign specimens (*P*=0.028, two-tailed Fisher exact test).

## DISCUSSION

Subtracted libraries have been constructed from well-characterised benign and malignant human breast tumour cell lines. The Huma 123, typical of human benign tumour cells, is oestrogen receptor negative and was compared with the less-malignant oestrogen receptor positive MCF-7 cell line, in preference to a more-malignant oestrogen receptor negative one such as MDA-MB-231, which lacks epithelial markers characteristic of breast carcinoma cells (Engel and Young, 1978). Although it was expected that oestrogen responsive cDNAs would be present in an MCF-7 library, the use of the better-defined breast epithelial MCF-7 cells reduced the likelihood of isolating cell-culture-related cDNAs. The libraries were discrete, and subtracted cloned cDNAs were differentially expressed when tested by reverse Northern and Northern hybridisation techniques. Although some cDNAs were differentially expressed by up to 36-fold, 76% and 83% of cDNAs in the benign and malignant libraries, respectively, were differentially expressed at a level of greater than two-fold. In both libraries, the majority of cloned cDNAs (66% and 56% for the benign and malignant subtracted libraries, respectively) represented products of previously-described genes. However, 28% of the sequenced clones in the benign cell library represented ESTs, but for the malignant library this proportion was somewhat higher at 41%, perhaps reflecting a greater number of previously-uncharacterised genes differentially-expressed in the malignant cells relative to the more tightly regulated cells of benign tumour origin. Only small proportions of the libraries were picked for sequencing. The observation that in each library, the majority of randomly picked cloned cDNAs were both differentially expressed and unique sequences suggests that these large libraries represent resources which contain further differentially-expressed cDNAs.

A recent study, using MCF-7 cells grown in the presence or absence of steroids as the starting material for subtraction, yielded 14 oestrogen responsive cDNAs (Ghosh *et al*, 2000). In another, the dissimilar oestrogen-receptor positive MCF-7 cell line and the oestrogen receptor negative, breast carcinoma cell line MDA-MB-231 yielded only 28 separate cDNAs (Kuang *et al*, 1998). In the present experiments, over 100 cDNAs have been isolated so far, possibly suggesting greater variation in gene expression between a benign and a malignant mammary cell line, than between two malignant mammary cell lines (Kuang *et al*, 1998). However, a small subset of cloned cDNAs found to be elevated in the present malignant library was also differentially-expressed in the previously-reported screen between the MCF-7 cells and MDA-MB-231 cells (Kuang *et al*, 1998), including cytokeratins 8 and 18 and CD 24 (Yang *et al*, 1999). Since the Huma 123 cell line and the MCF-7 cell lines used in the present study have the same oestrogen receptor status as the MDA-MB-231 cells and the MCF-7 respectively (Kuang *et al*, 1998), it is likely that the mRNAs represented by these cloned cDNAs, are associated with the differing oestrogen receptor status of the presently-used cell lines. Furthermore, other ER-dependent mRNAs, including Cyclin D1 (Altucci *et al*, 1996), pS2 (Masiakowski *et al*, 1982) and pLIV-1 (Manning *et al*, 1988), were also present in the malignant subtracted library.

cDNAs from the present libraries that have been described previously suggest that the libraries do contain cDNAs with relevance in the development of breast cancer. For example, over-expression of Cyclin D1 has been shown to distinguish breast carcinomas and *in situ* breast lesions from benign lesions (Weinstat-Saslow *et al*, 1995). Amongst other cDNAs up-regulated in the malignant cell library, were those corresponding to mRNAs for a putative G-protein-coupled receptor (Libert *et al*, 1989), for proteins associated with the mitotic spindle checkpoint, BUB3 (Brady and Hardwick, 2000) and with chromatin remodelling (Wang *et al*, 1996; Zhang *et al*, 1998; Vignali *et al*, 2000). Two cDNAs (LIM kinase and BUB3), which are differentially-expressed in the present malignant library, have also been found to be differentially expressed in a subtracted library representing cDNAs expressed in a pathologically homogeneous breast carcinoma specimen subtracted with cDNA from 50 000 ductal-carcinoma-*in-situ* cells microdissected from the surrounding normal tissue (Liu, 2001). Although the dependence of expression of the mRNAs for these cloned cDNAs on oestrogen is not known, the present work clearly identifies many relevant cloned cDNA targets for further investigation.

The subtracted library of cDNAs down-regulated in the MCF-7 cells relative to the Huma 123 cells contained a cDNA corresponding to the mRNA for an EF-hand protein, S100A2, which was shown previously to be abundantly expressed in both normal breast and benign breast tumour specimens (Liu *et al*, 2000), but only detectable in less than 15% of malignant breast cancer specimens tested. This result suggests that the suppression subtractive hybridisation produces libraries containing cDNAs differentially expressed between benign and malignant cells.

The library of cDNAs up-regulated in MCF-7 cells relative to the benign Huma 123 cells contained ESTs that exhibited oestrogen dependence. One such cloned cDNA, M41, corresponds to a transcript of a previously unmapped gene located on chromosome 21q22.3. 5′ and 3′ RACE experiments showed that M41 mRNA possesses a complex pattern of spliced variants in the MCF-7 cells, some of which contain short open-reading-frames which can be translated into protein using a coupled *in vitro* transcription translation system (not shown). However, the splice variants were not sufficiently different in size to be individually distinguishable in the Northern blotting experiments. The individual introns and exons of this new gene have now been precisely mapped onto chromosome 21 contigs.

M41 mRNA was shown to be produced by the carcinoma cells of breast cancer specimens using *in situ* hybridisation, and, using a PCR assay, M41 mRNA was found not to be statistically significantly correlated with the presence of oestrogen receptor, nor with the immunocytochemical detection of the oestrogen-responsive progesterone receptor (not shown) in a panel of carcinoma specimens. Thus, although M41 mRNA relates to oestrogen receptor status in the malignant breast-derived cell lines tested, in the carcinoma specimens, the relationship is stronger between benign and malignant than between oestrogen receptor positive and negative. These results probably relate to the complexity of oestrogen receptor regulation of gene expression. It is the results with the carcinoma specimens that are likely to be more important. A similar weakening of correlation with ER status of carcinoma specimens has been found for two different cDNAs, pDZK1 and GREB1, which also showed a strong correlation with ER in cell lines (Ghosh *et al*, 2000), but which exhibited a much weaker correlation with oestrogen receptor positivity in carcinoma specimens. This lack of correlation between oestrogen responsive cDNAs in MCF-7 cells and carcinoma specimens, and the variability of patterns of gene expression in breast carcinomas has recently also been reported using array technology (Gruvberger *et al*, 2001). The M41 gene contains an oestrogen response element associated with an intronic *alu* repeat sequence. Such variant *alu*-DNA repeats, that can act as oestrogen and other steroid hormone response elements, have been described previously (Norris *et al*, 1995; Babich *et al*, 1999). The M41 gene *alu-*associated ERE is of the inverted repeat type with a 17 nucleotide separation (IR-17) as described by Babich *et al* (1999). Whilst it is known that similar elements can activate reporter gene activity in a hormone dependent manner in cultured cells (Norris *et al*, 1995; Babich *et al*, 1999) and also bind proteins *in vitro* from nuclear extracts of cultured cells (Babich *et al*, 1999), little is known of their activity in carcinoma cells that have not been subjected to cell culture. In the present experiments on carcinoma specimens, the statistically-significant difference in the expression of M41 mRNA between malignant human breast specimens and either benign or benign and normal breast specimens, but the lack of correlation with oestrogen receptor status, suggests that M41 mRNA is up-regulated during the malignant progression of carcinomas, by an oestrogen-independent mechanism.
